# OrthoRefine: automated enhancement of prior ortholog identification via synteny

**DOI:** 10.1186/s12859-024-05786-7

**Published:** 2024-04-25

**Authors:** J. Ludwig, J. Mrázek

**Affiliations:** 1 Institute of Bioinformatics, The University of Georgia, Athens, GA 30602 USA; 2 Department of Microbiology and Institute of Bioinformatics, The University of Georgia, Athens, GA 30602 USA

**Keywords:** Ortholog, Synteny, Homolog, Paralog, Orthogroup

## Abstract

**Background:**

Identifying orthologs continues to be an early and imperative step in genome analysis but remains a challenging problem. While synteny (conservation of gene order) has previously been used independently and in combination with other methods to identify orthologs, applying synteny in ortholog identification has yet to be automated in a user-friendly manner. This desire for automation and ease-of-use led us to develop OrthoRefine, a standalone program that uses synteny to refine ortholog identification.

**Results:**

We developed OrthoRefine to improve the detection of orthologous genes by implementing a look-around window approach to detect synteny. We tested OrthoRefine in tandem with OrthoFinder, one of the most used software for identification of orthologs in recent years. We evaluated improvements provided by OrthoRefine in several bacterial and a eukaryotic dataset. OrthoRefine efficiently eliminates paralogs from orthologous groups detected by OrthoFinder. Using synteny increased specificity and functional ortholog identification; additionally, analysis of BLAST e-value, phylogenetics, and operon occurrence further supported using synteny for ortholog identification. A comparison of several window sizes suggested that smaller window sizes (eight genes) were generally the most suitable for identifying orthologs via synteny. However, larger windows (30 genes) performed better in datasets containing less closely related genomes. A typical run of OrthoRefine with ~ 10 bacterial genomes can be completed in a few minutes on a regular desktop PC.

**Conclusion:**

OrthoRefine is a simple-to-use, standalone tool that automates the application of synteny to improve ortholog detection. OrthoRefine is particularly efficient in eliminating paralogs from orthologous groups delineated by standard methods.

**Supplementary Information:**

The online version contains supplementary material available at 10.1186/s12859-024-05786-7.

## Background

Discerning the evolutionary relationship between genes and genomes remains a fundamental step in the search for answers to many biological questions. The need for accurate assessment of evolutionary relationships among genes is inherent to many different tasks: whether the goal is the construction of phylogenetic trees [1], using orthologs to infer the unknown function of a hypothetical gene [2–5], constructing databases for functional and comparative genomics [6, 7], verifying genome assemblies [8], or understanding the principles of genome organization and evolution [9–11]. However, the accurate identification of evolutionary relationships between genes can be confounded by evolutionary events, such as gene or genome duplication, gene loss, and gene acquisition via horizontal gene transfer [12]. Furthermore, automating the ortholog identification process is challenging, although not intractable [13].

In the context of gene history and comparisons, genes that evolved by divergence from a shared ancestor are classified as homologs [1, 14]. Gene homology can be further divided into three types based on the events that allowed the genes to take different evolutionary paths: (1) orthologs diverged because of a speciation event, (2) paralogs diverged following a gene duplication event [1], and (3) xenologs arose due to a horizontal gene transfer event [15]. To relate gene duplication events with speciation events, paralogs can be further classified as inparalogs, those that arose from a duplication event after the speciation event, and outparalogs, those already present before the speciation event [16].

While all genes experience genetic changes, orthologs tend to retain the function of the shared ancestral gene more often than paralogs [17, 18]; this property of orthologs is routinely used to predict functions of genes that are yet to be experimentally characterized. Another application of gene orthology is in judging the quality of de novo genome assemblies by verifying that they have the expected single-copy orthologs present in other related species [8]. Regardless of the application, the core assumption is that orthologous genes tend to retain their function after divergence, which is less likely for paralogs or xenologs.

Current informatics-based methods for the identification of orthologs are rooted in either phylogenetics, as is the case with Ortholuge which compares ratios of phylogenetic distances between ingroups and an outgroup to improve the accuracy of prior ortholog identification [19], or reciprocal best hit (RBH) via BLAST combined with a clustering algorithm—e.g., OrthoMCL performs Markov clustering on RBH results [20]. Other ortholog identification programs include OrthoLoger [21], TreeFam [22], and InParanoid [23]. However, the original software that implemented these methods is generally no longer functional on present-day computers and operating systems due to dependencies on obsolete versions of software components or the software is challenging to set up and use [24].

An alternative but often not an ideal solution for the identification of orthologs is to use one of several available databases of orthologous genes, such as OrthoDB [25], eggNOG [26], PANTHER [27], or OMA [28]. This solution requires that the database contains information on the genome of interest and that the data from the database is in an accessible format that can be used for analysis. In addition, the databases generally do not allow changing the parameters of the algorithm used to identify the orthologs (e.g., sequence similarity cutoff, clustering parameters, phylogenetic models and their parameters), and the default parameters may not be ideal for different types of studies [29].

OrthoFinder combines reciprocal best-hits with phylogenetics to identify orthologs. The major advantages over other software include that it is user-friendly, easy to install (it does not rely on additional software not included in the installation), and offers increased accuracy for ortholog identification compared to many earlier methods [13, 24]. In OrthoFinder’s 2015 paper, the authors noted the possibility of using synteny (conservation of gene order) to refine ortholog identification. However, they chose not to use synteny because reliable syntenic information breaks down over long evolutionary distances and syntenic information is not immediately available for de novo assemblies. We note that for de novo assemblies, a genome annotation (feature table file) may be obtained by submitting the data for automated annotation at NCBI, or it can be directly generated by the user using the same pipeline [30]. Moreover, our results below, and other recent work [31], show that ortholog identification via OrthoFinder can be enhanced by using synteny information.

The term synteny was initially conceived to describe genes linked together during inheritance (chromosome mapping, see [32]) and referred to two or more genes located on the same chromosome [33]. More recently, particularly in the context of prokaryotic genomes, the term “synteny” has been used in reference to conserved gene order in comparative genomics [5, 34, 35]. This is how we use the term “synteny” in this work.

OrthoFinder is an effective tool that provides results suitable for many tasks. Nonetheless, incorporating synteny into the criteria for identifying orthologs as an additional postprocessing step can enhance the program’s ability to distinguish orthologs from paralogs and further refine some of the HOGs (hierarchical orthogroups) reported by OrthoFinder. Here we present a new program, OrthoRefine, which automates the task of using synteny information to refine the HOGs identified by OrthoFinder into groups of syntenic orthologs, orthologs grouped based on evidence of synteny. We expect this tool to be used primarily in tasks that would benefit from resolving orthologous relationships to no more than a single ortholog from each compared genome in each orthologous group. OrthoRefine was designed to emulate several of the qualities that make OrthoFinder a desirable tool for the end user: speed, ease-of-use, and self-containment (no dependencies); OrthoRefine requires only the output from OrthoFinder and genome annotations (in the RefSeq features table format used by NCBI) and does not depend on any other software or data that could complicate its use. The only input required to be created by the end user is a text file where each line specifies the Refseq accession for each genome used as input for OrthoFinder. While we used OrthoRefine in combination with OrthoFinder, it can refine the output of other programs that provide an initial clustering of homologous genes if the output is formatted to match OrthoFinder’s.

## Implementation

### OrthoFinder Summary

OrthoFinder was initially described in 2015 [24], and updated software and manuscript were released four years later [13]; a subsequent update in version 2.4.0 introduced a new final output in the form of hierarchical orthogroups (HOGs) [36]. (The original orthogroups were defined as all genes predicted to be descendants of a single gene of the last common ancestor, not distinguishing orthologs from paralogs. HOGs are expected to be more accurate in identifying orthologs than the orthogroups.) We used version 2.5.2, downloaded from the GitHub repository on April 6th, 2021.

While there are options and parameter adjustments that may be used when running OrthoFinder (e-value of BLAST, MCL inflation parameter, and phylogenetic parameters, etc.), our focus was on running OrthoFinder with default settings because, in our review of literature citing OrthoFinder, OrthoFinder was generally used with default parameters.

### Identification of syntenic ortholog groups using a look-around window (OrthoRefine algorithm description)

**Fig. 1:**
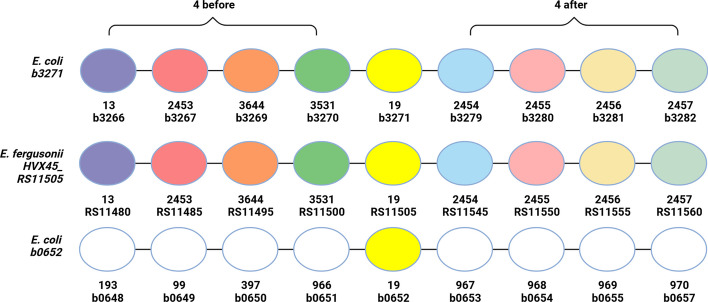
Example of OrthoRefine’s synteny analysis. The window around three genes assigned to HOG19 by OrthoFinder demonstrates how OrthoRefine determines which of the *E. coli* genes is an ortholog of *E. fergusonii’s* HVX45_RS11505. The HOG19 genes are shown with yellow fill, other genes assigned to the same HOG are shown in matching colors, and genes that have orthologs in other genomes outside the displayed window are shown in white. The first number below each circle denotes the HOG assigned by OrthoFinder, while the second entry shows the locus tag

By default, OrthoRefine is only applied to HOGs with at least two genomes and at least two genes from the same genome (paralogs), with an option to verify synteny for HOGs that have only a single gene from each genome. The latter may still include paralogs if genes were duplicated and the original copy was subsequently lost, which can be revealed by synteny. The analysis begins by constructing a window centered at each gene of the HOG. OrthoRefine evaluates the synteny by counting matching pairs of genes inside the window; matching pairs consist of genes assigned to the same HOG in the initial OrthoFinder output (Fig. 1). We note that genes only need to be within the window and are not required to be in the same order, and genes that do not have a homolog in the other genome are not included in the window (see Fig. 5 for a visual explanation). The synteny ratio, sr, is calculated by taking the number of matching pairs and dividing it by the window size, w (Eqs. 1). If the ratio is greater than a cutoff (default 0.5), the genes at the center of the window are considered syntenic. After a pairwise comparison between all genes of different genomes in the original HOG, any subset of genes linked by synteny is referred to as a syntenic ortholog group (SOG). A HOG can thus be refined into one SOG (by removing paralogs which do not exhibit synteny with any other genes from the original HOG) or into more than one SOG if the original HOG contained multiple distinct subgroups of genes linked by synteny within each subgroup but not between the subgroups.

1 $${x}_{i}=\left\{\begin{array}{ll}1& if\; match\\ 0& if \; no\, match\end{array}\right., sr=\frac{{\sum }_{1}^{w}{x}_{i}}{w}$$

where sr is the synteny ratio, w is the window size, and i is the serial number of the gene within the window. The gene being evaluated for synteny (at the center of the window) is not counted.

### Runtime parameters

Two runtime parameters control OrthoRefine, window size and synteny ratio. There is no consensus on the required amount of synteny—how many surrounding genes in a window must be orthologs to conclude that the gene of interest is an ortholog—or the size of the window. We recommend a smaller window size and larger synteny ratio when analyzing datasets containing closely related genomes, e.g., window size eight and synteny ratio 0.5. In contrast, a larger window size and or a lower synteny ratio may be more appropriate as the evolutionary distance increases, e.g., window size 30 and synteny ratio 0.2. After testing several combinations of window size and synteny ratio, we selected a window size of eight and synteny ratio of 0.5 as default parameters (see Results and Discussion for details).

### OrthoRefine application example (Four *Escherichia* genomes)

We demonstrate the use of OrthoRefine on HOG 19 from the OrthoFinder output for representative genomes of four different species of the *Escherichia* genus: *Escherichia coli* strain K12 substrain MG1655 (NCBI genome assembly GCF_000005845.2), *Escherichia fergusonii* (GCF_013892435.1), *Escherichia albertii* (GCF_016904755.1), and *Escherichia marmotae* (GCF_902709585.1). OrthoFinder, with default parameters, was used to identify HOGs, and OrthoRefine was subsequently applied with window size eight and synteny ratio cutoff 0.5.

The HOG included four *E*. *coli* genes (b0652, b3271, b4106, & b4096), fives genes from *E*. *fergusonii* (HVX45_RS09410, HVX45_RS02390, HVX45_RS04025, HVX45_RS07420, & HVX45_RS11505), two genes from *E*. *marmotae* (GV529_RS14465 & GV529_RS05870), and one gene from *E*. *albertii* (JRC41_RS15115). Most of the genes were annotated as encoding ATP-binding cassette (ABC) transporters. Figure 1 shows how OrthoRefine determined which of b3271 or b0652 of *E. coli* is the ortholog of RS11505 of *E. fergusonii.* As eight out of eight genes surrounding RS11505 had a match in the window centered at b3271, we concluded that there is a syntenic relationship between *E*. *coli’s* b3271 and *E*. *fergusonii’s* RS11505, and they are orthologs while b0652 is presumed to be a paralog of RS11505; none of the genes surrounding b0562 had a match within the window around RS11505. This HOG was ultimately refined into two SOGs: the first included a single syntenous ortholog from each genome and the second SOG contained a single syntenous ortholog from *E*. *coli*, *E. fergusonii*, and *E. marmotae*. The remaining genes initially placed in HOG 19 by OrthoFinder were excluded by OrthoRefine as putative paralogs (see Results and Discussion for details).

## Results and discussion

### Datasets used to evaluate OrthoRefine’s performance

We analyzed several datasets including taxa of different levels of divergence. The first dataset, Quest for Orthologs [37], included 23 diverse bacterial genomes used to test OrthoRefine using the community standard benchmarking tool [38]. The second dataset was the four *Escherichia* species detailed above. The third dataset comprising four Gammaproteobacteria—*E. coli, Klebsiella pneumoniae*, *Salmonella enterica*, and *Pseudomonas aeruginosa*—was adopted from a prior study [39]. The fourth dataset was a collection of sixteen members of the phylum Actinomycetota. The fifth dataset was used to test OrthoRefine’s performance with eukaryotic genomes; three *Saccharomyces* genomes were selected: *Saccharomyces mikatae*, *Saccharomyces cerevisiae*, and *Saccharomyces kudriavzevii*.

### Benchmarking OrthoRefine

Orthology Benchmarking [38], a web-based benchmarking tool, was used to evaluate OrthoRefine’s ability to improve functional ortholog identification (gene ontology conservation (GO) & enzyme classification (EC); [40]) and specificity (Robinson-Foulds (RF) distance; [40]). Because the Refseq annotations and sequences corresponding to the date when the benchmarking data were generated (2020) are no longer available on the NCBI website (https://ftp.ncbi.nlm.nih.gov/genomes/all/GCF/), we were unable to use precisely the same collection of proteins that was used for the benchmarking (the annotations are required to determine gene location to assess synteny) and our results are not directly comparable to the data on the benchmarking server. However, we utilized the benchmarking tool to compare OrthoFinder and OrthoRefine results using a dataset that was composed of the same 23 bacterial genomes (Table 1) as the benchmarking dataset but with current annotations and protein sequences downloaded in August 2023 (see Additional file 1 for details on generating the dataset). This dataset includes genomes spanning diverse bacterial phyla and the large evolutionary distances among the genomes makes synteny less effective, providing a stringent test for OrthoRefine.

**Table 1:** Names and RefSeq accessions for the 23 genomes from the Quest for Orthologs

Genius species	RefSeq accession
*Mycobacterium tuberculosis*	GCF_000195955.2
*Pseudomonas aeruginosa*	GCF_000006765.1
*Thermotoga maritima*	GCF_000008545.1
*Chlamydia trachomatis*	GCF_000008725.1
*Streptomyces coelicolor*	GCF_000203835.1
*Leptospira interrogans*	GCF_000092565.1
*Escherichia coli*	GCF_000005845.2
*Neisseria meningitidis*	GCF_000008805.1
*Deinococcus radiodurans*	GCF_000008565.1
*Bradyrhizobium diazoefficiens*	GCF_000011365.1
*Synechocystis*	GCF_000009725.1
*Chloroflexus aurantiacus*	GCF_000018865.1
*Bacillus subtilis*	GCF_000009045.1
*Gloeobacter violaceus*	GCF_000011385.1
*Aquifex aeolicus*	GCF_000008625.1
*Helicobacter pylori*	GCF_000008525.1
*Fusobacterium nucleatum*	GCF_000007325.1
*Rhodopirellula baltica*	GCF_000196115.1
*Geobacter sulfurreducens*	GCF_000007985.2
*Mycoplasma genitalium*	GCF_000027325.1
*Dictyoglomus turgidum*	GCF_000021645.1
*Bacteroides thetaiotaomicron*	GCF_000011065.1
*Thermodesulfovibrio yellowstonii*	GCF_000020985.1

**Fig. 2:**
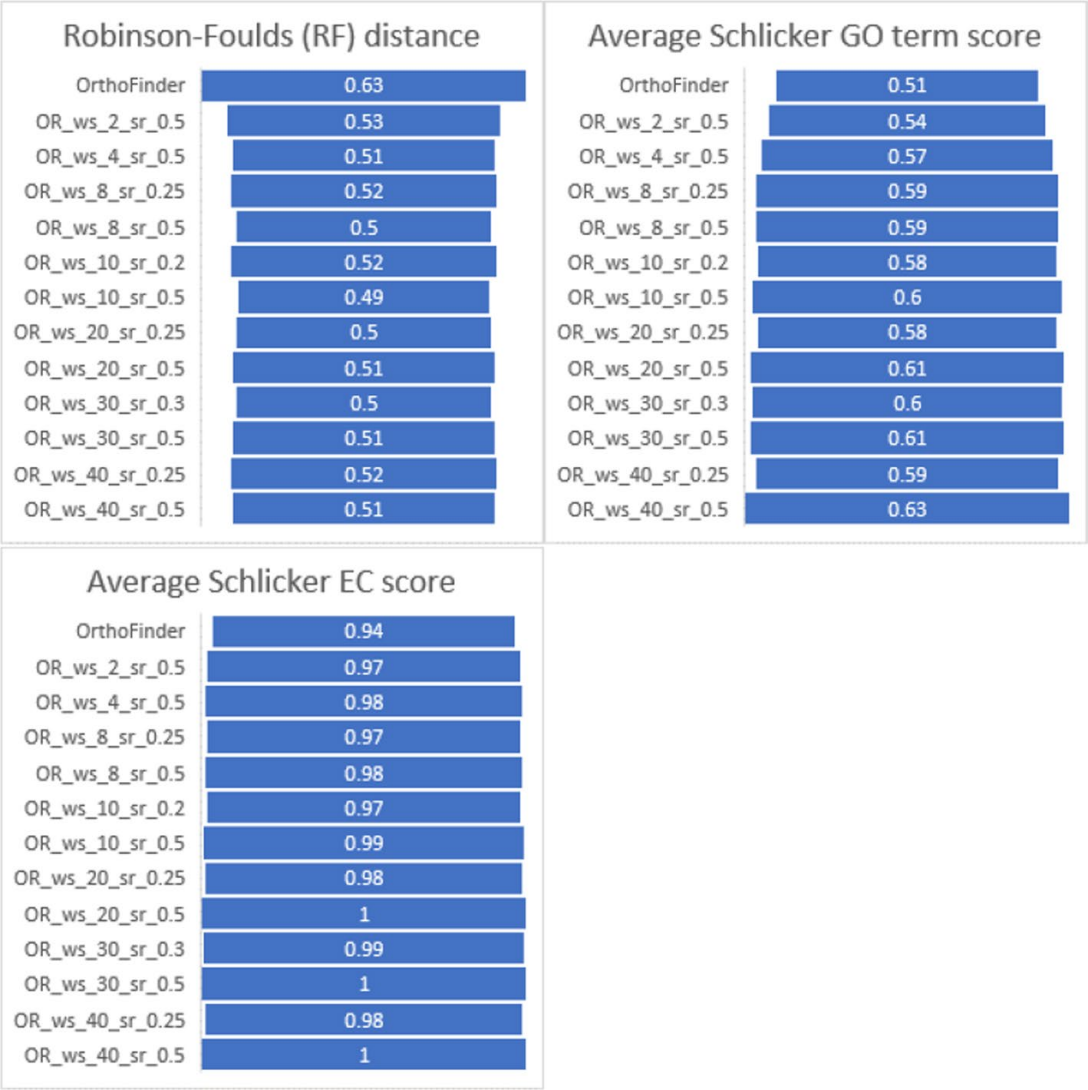
Benchmarking results for OrthoFinder and OrthoRefine on the Quest for Orthologs bacterial dataset. OrthoRefine was run with different parameters for window size (ws) and synteny ratio (sr). **A** Robinson-Foulds (RF) distance as a measure of specificity (lower values indicate higher specificity). **B** Average Schlicker scores for gene ontology (GO) and **C** enzyme classification (EC) as a measure of functional ortholog identification (higher scores indicate improvement)

As expected, OrthoRefine increased functional ortholog identification and specificity accuracy compared to OrthoFinder alone. Of the combinations for window size and synteny ratio we tested, window size ten and synteny ratio 0.5 resulted in the lowest RF distance (conceptually defined as a normalized sum of differences between the benchmark orthogroups and the user proposed orthogroups; a lower score indicates higher specificity). The highest average Schlicker score [41] (a measure of the overall mutual similarity of the function classifications of the orthologs) for the GO terms was observed at window size 40 and synteny ratio 0.5, while the highest average Schlicker score for EC was recorded for window sizes 20–40 at a synteny ratio of 0.5 (Fig. 2).

### Evaluating OrthoRefine’s runtime parameters (window size & synteny ratio)

In the absence of a gold standard dataset of true orthologs to assess OrthoRefine’s accuracy, we consider that, for most applications, the most desirable set of orthologs would include genes from the maximum number of the genomes analyzed (high sensitivity) while containing no paralogs (i.e., a single gene from each genome; high specificity). We, therefore, evaluated OrthoRefine’s output for the average maximum number of orthologous genes (AMNOG) defined as the dataset’s average maximum number of orthologs present in SOGs without paralogs. For HOGs that were refined into multiple SOGs, the SOG with the most genomes represented is included in the AMNOG calculation. We propose to use the AMNOG as a relative measure of sensitivity while specificity is, in theory, at or near 100% (SOGs containing paralogs are excluded). In general, changing parameters did not dramatically change the AMNOG measure. We observed that larger windows and lower synteny ratio performed slightly better in datasets consisting of diverse genomes. For datasets of closely related genomes, smaller window sizes performed at least equally as well as larger window sizes and within those smaller window sizes, a larger synteny ratio tended to result in a higher AMNOG (Table 2).

**Table 2:** Combinations of window size and synteny ratio on the AMNOG

Window size	Synteny ratio	4 *Escherichia*	4 Gammaproteo-bacteria	16 Actino-mycetota	3* Saccharomyces*	Average AMNOG
		Average max number orthologous genes (AMNOG)				
2	0.5	3.16	2.52	3.2	2.3	2.8
4	0.25	3.15	2.54	3.21	2.33	2.81
4	0.5	3.2	2.57	3.41	2.44	2.91
6	0.2	3.17	2.6	3.41	2.32	2.88
6	0.5	3.19	2.59	3.38	2.55	2.93
8	0.2	3.19	2.57	3.42	2.41	2.9
8	0.3	3.2	2.57	3.44	2.54	2.94
8	0.5	3.24	2.57	3.39	2.59	2.95
10	0.2	3.22	2.58	3.4	2.39	2.9
10	0.3	3.22	2.57	3.45	2.6	2.96
10	0.5	3.23	2.56	3.34	2.67	2.95
30	0.2	3.22	2.6	3.4	2.72	2.99
30	0.3	3.23	2.56	3.31	2.73	2.96
30	0.5	3.17	2.54	3.02	2.68	2.85
40	0.2	3.2	2.59	3.44	2.73	2.99
40	0.3	3.21	2.57	3.29	2.69	2.94
40	0.5	3.18	2.53	2.9	2.7	2.83

### Testing OrthoRefine on datasets of varying phylogenetic diversity

#### *Escherichia* dataset

**Fig. 3:**
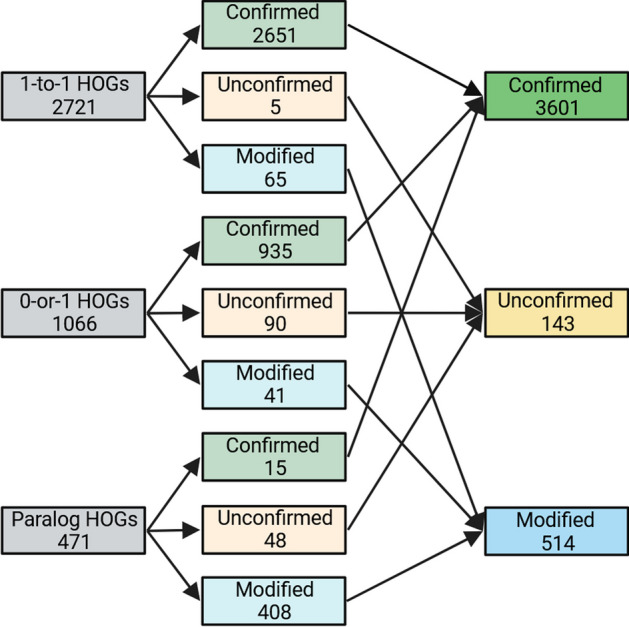
Summary statistics for the four *Escherichia* genomes. The genomes were analyzed with OrthoRefine (window size = 8; synteny ratio = 0.5). 1-to-1 HOGs contained precisely one gene per genome. 0-or-1 HOGs were missing an ortholog in at least one genome and none of the genomes contributed more than one gene. Paralog HOGs are those where at least one genome contributed more than one gene. Confirmed HOGs are those where all genes assigned by OrthoFinder to a HOG were supported by synteny. Unconfirmed HOGs lacked synteny support for all genes assigned to a HOG by OrthoFinder or any SOG subgroup. HOGs where synteny eliminated at least one paralog and/or divided the HOG into at least one SOG are designated Modified HOGs. HOGs comprised of genes of only one genome could not be analyzed by OrthoRefine and were excluded

We evaluated OrthoRefine on four species of the *Escherichia* genus. The close relationship among the genomes was expected to make accurate identification of orthologs easier for OrthoFinder and OrthoRefine due to the low divergence between orthologous gene sequences and the high conservation of gene order. Indeed, 64% of OrthoFinder’s HOGs were comprised of precisely one gene from each genome (1-to-1 HOGs), while 25% of the HOGs were missing an ortholog in at least one genome but included no more than one gene per genome (0-or-1 HOGs). 11% of HOGs combined orthologs and paralogs (at least one genome contributed more than one gene to the same HOG). OrthoRefine modified 87% of these paralogous HOGs (synteny eliminated at least one paralog and or divided the HOG into at least one SOG); the remaining 13% were split between either confirmed (all genes assigned by OrthoFinder to a HOG were supported by synteny) (3%) or unconfirmed (insufficient synteny support for the original HOG or any SOG subgroup) (10%). Additionally, OrthoRefine confirmed 97% of the 1-to-1 HOGs and 88% of the 0-or-1 HOGs (Fig. 3).

**Case study 1: HOG19 – (ABC transporters)** HOG19, identified by OrthoFinder, contained a group of genes encoding ATP-binding cassette (ABC) transporters. ABC transporters are easily identifiable by the presence of the distinctive ATP-binding domain, but their further classification remains challenging, in part, due to the vast diversity of substrates they can transfer and the often subtle differences that can affect substrate specificity [42].

**Fig. 4:**
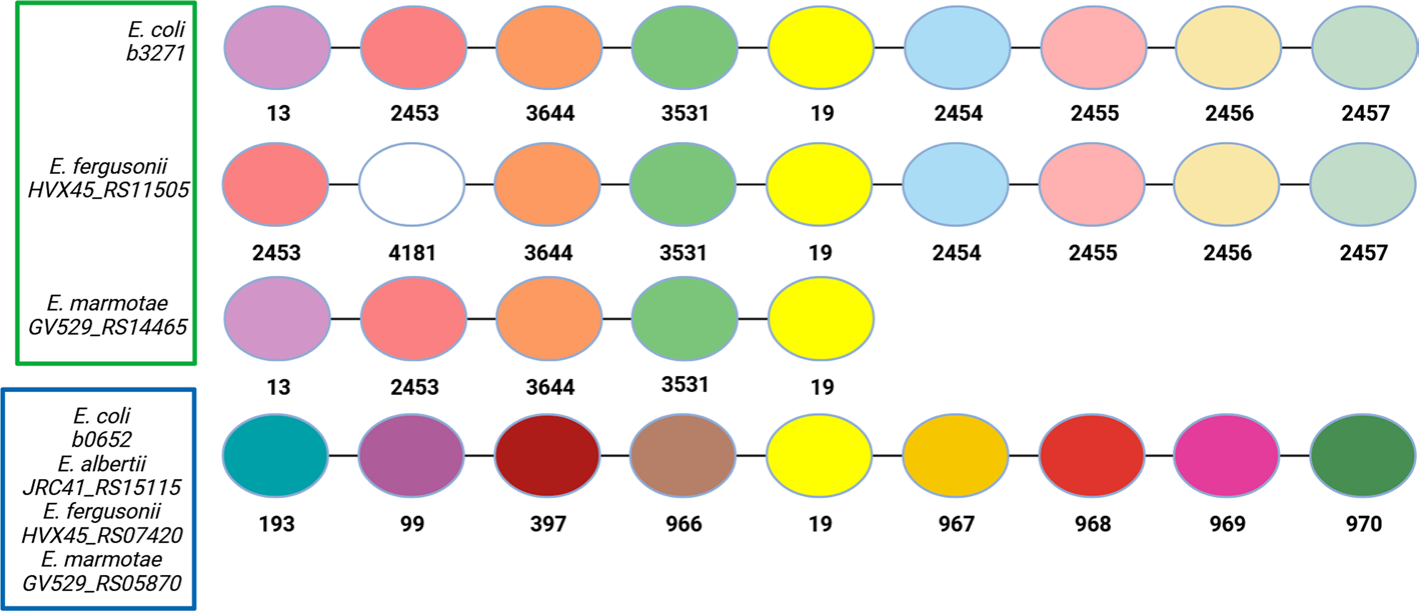
Synteny analysis of HOG 19. Matched colored circles represent genes assigned to the same HOG by OrthoFinder, with the HOG numbers shown below each circle; white circles denote genes with orthologs in other genomes located outside the displayed window. Green and blue boxes mark the two SOGs delineated by OrthoRefine. The missing data from *E. marmotae* to the right of the HOG19 member is due to a scaffold boundary in the assembly. The neighborhoods for genes marked by the blue box are identical and have been collapsed into a single line. The other members of HOG19 (b4106, b4096, HVX45_RS02390, HVX45_RS04025, & HVX45_RS09410) are omitted because they have no syntenic matches to any other member of HOG19

OrthoRefine divided this HOG into two subgroups: SOG19.0 which consisted of b0652, JRC41_RS15115, HVX45_RS07420, & GV529_RS05870 and SOG19.1 which consisted of b3271, HVX45_RS11505, & GV529_RS14465, while excluding b4106, b4096, HVX45_RS02390, HVX45_RS04205, & HVX45_RS09410 as presumed paralogs (Fig. 4). The BLAST e-values and percent identity from OrthoFinder’s alignment supported the presence of these two natural subgroups in HOG19 (Additional file 2: Table S1), as did the phylogenetic tree made independently of OrthoFinder (Additional file 3: Figure S1) using Muscle, version 5 [43], RAxML, version 8.2.12 [44], R, version 4.1.2 [45], and the R library ape, version 5.6–2 [46] (see Additional file 4 for commands used). Previously reported operon structures further supported the division of HOG19 by OrthoRefine (Additional file 3: Figure S2).

**Case study 2: HOG21 – (*rpnA*/*rpnE* homologs)** HOG 21 comprised eight genes from the four genomes; the genes were annotated as encoding recombination-promoting nuclease (*rpnA*), *rpnE*, insertion sequence family not classified yet (ISNCY), or hypothetical protein. The recombination-promoting nucleases are thought to be involved with horizontal gene transfer, though RpnE was inactive in recombination-determining assays [47].

**Fig. 5:**
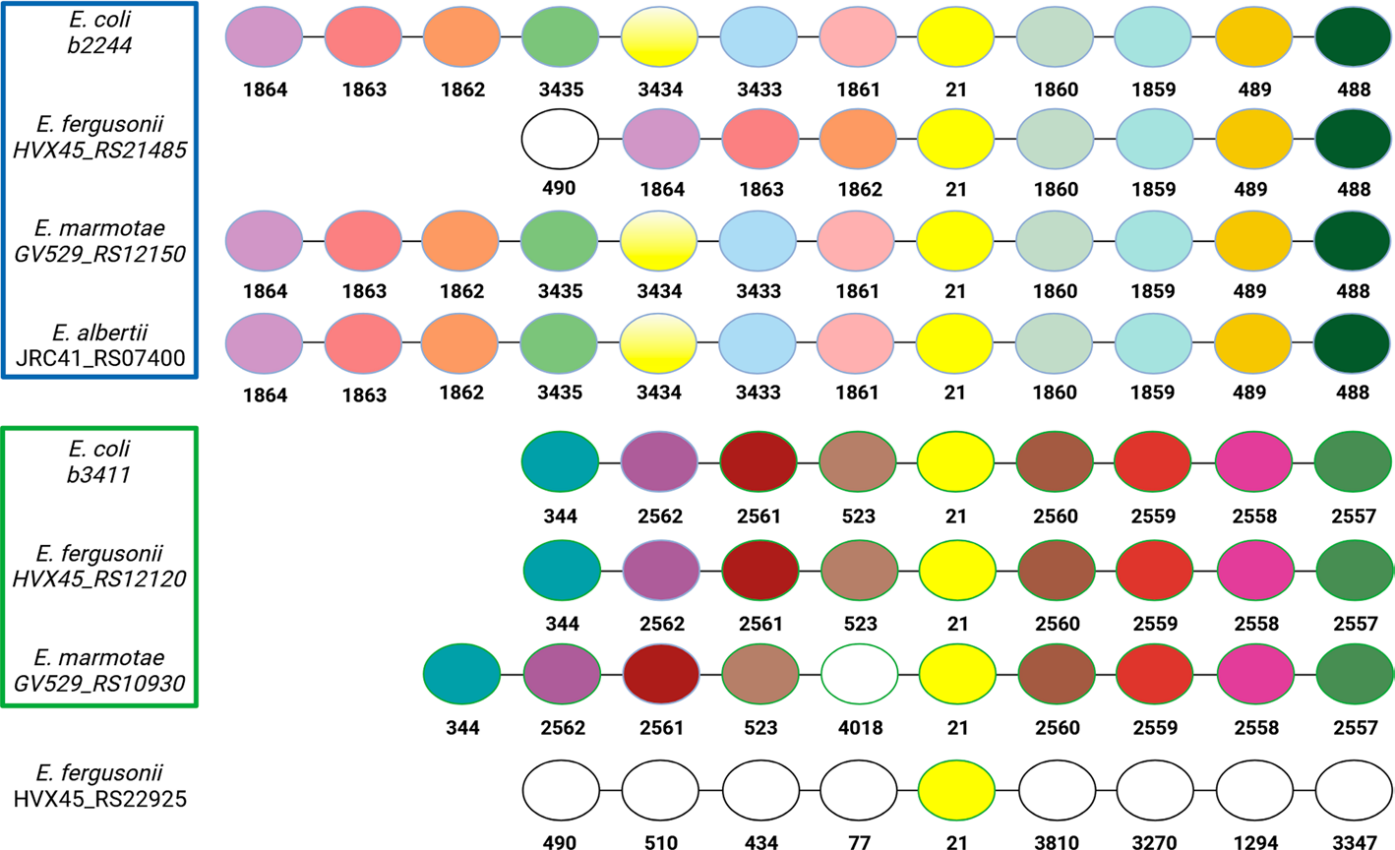
Synteny analysis of HOG 21. Matched colored circles represent genes assigned to the same HOG by OrthoFinder, which is shown below each circle; white circles denote genes with orthologs in other genomes located outside the displayed window. Green and blue boxes mark the two SOGs identified from HOG21. The analysis was performed with window size 8 (four genes on each side of HOG21). However, because the synteny is evaluated separately for each pair of genomes and the *E. fergusonii* genome contains no representative of HOGs 3433, 3434, and 3435, these genes are excluded from comparisons with *E. fergusonii* (OrthoRefine ignores genes that do not have a counterpart in the other genome) and the window instead includes an additional three genes (HOGs 1862, 1863, and 1864), which allows HVX45_RS21485 to be identified as the syntenous ortholog of b2244, GV529_RS12150, and JRC41_RS07400

Similar to HOG19 above, OrthoRefine divided HOG21 into two subgroups: SOG21.0 – the *rpnE* and ISNCY group, which consisted of b2244, HVX_RS21485, GV529_RS12150, & JRC41_RS07400 and SOG21.1 – the *rpnA* group, which consisted of b3411, HVX45_RS12120, & GV529_RS10930 while excluding HVX45_RS22925 as a presumed paralog (Fig. 5). The BLAST e-values and percent identity supported dividing HOG21 into these two natural subgroups (Additional file 2: Table S2); additionally, the phylogenetic tree agreed with the two subgroups but included HVX45_RS22925 in the *rpnA* group (Additional file 3: Figure S3). The HVX45_RS22925 gene encodes a short (68 amino acids) hypothetical protein that is similar to the C-terminal segment of RpnA, which is, however, much larger (292 amino acids in *E. coli*). The similarity probably leads to this hypothetical protein being included in HOG21 by OrthoFinder, but its short length suggests that it is not a true ortholog of RpnA—if it is a functional protein at all.

### Gammaproteobacteria dataset (Lim et al. 2022)

**Fig. 6:**
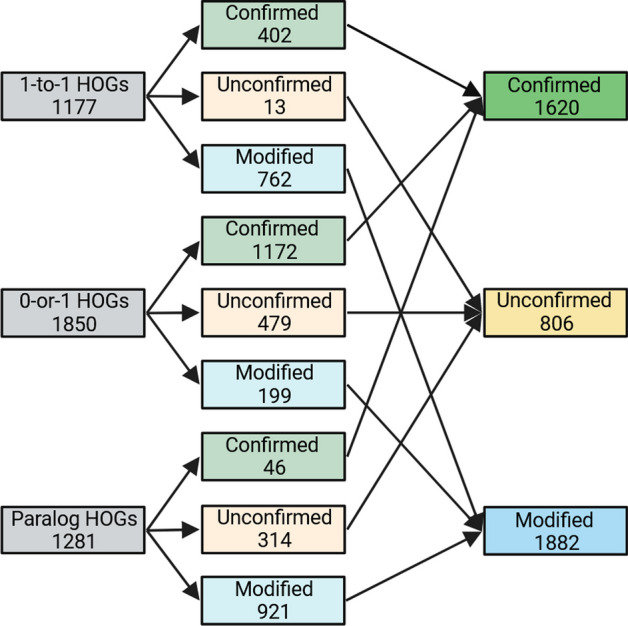
Summary statistics for the four Gammaproteobacteria genomes. The genomes were analyzed with OrthoRefine (window size = 8; synteny ratio = 0.5). See legend to Fig. 3

In their publication, the authors analyzed the dataset (Table 3) with OrthoFinder and highlighted a specific HOG which contained paralogs; we analyzed the same genomes with OrthoFinder and OrthoRefine to resolve the paralog HOG to a 1-to-1 relationship. We observed a lower percentage of 1-to-1 HOGs (27%) than in the *Escherichia* dataset, presumably due to the larger evolutionary distance among the genomes. The percentage of 0-or-1 HOGs (43%) and HOGs with paralogs (30%) increased. When using OrthoRefine to process OrthoFinder results, we observed a lower percentage of 1-to-1 HOGs (34%) and 0-or-1 HOGs (63%) confirmed by synteny and generally higher number of HOGs that were modified by OrthoRefine (Fig. 6).

**Table 3:** Names and RefSeq accessions for genomes used in Lim et al. 2022

Genius species	RefSeq accession	Gene locus tag (gene annotation)
*Escherichia coli*	GCF_000005845.2	b1916 (*sdiA*)
*Salmonella enterica*	GCF_000006945.2	STM1950 (*sdiA*)
*Klebsiella pneumoniae*	GCF_000445405.1	N559_RS09495 (*sdiA*)
*Pseudomonas aeruginosa*	GCF_001181725.1	AFI95_RS32400 (tr, *luxR* family) AFI95_RS29375 (*lasR*) AFI95_RS07465 (*rhlR*) AFI95_RS28195 (*qscR*)

Gene annotations are in parenthesis: Suppressor of cell division A (*sdiA*), transcription regulator (tr) which is further noted as part of the luminescence (*luxR*) family, regulator of elastase lasB (*lasR*), rhamnolipid regulator (*rhlR*), and quorum-sensing transcription repressor (*qscR*)

**Case study 3: HOG346—(*sdiA*)** *sdiA* encodes a LuxR family transcription factor and is thought to regulate transcription of cell division genes [48] and genes involved in acid tolerance [49]. OrthoFinder included potential paralogs in this HOG in the original analysis by Lim et al. ([39] Fig. 2 C & D) and the same genes were included in this HOG in our own results when we used OrthoFinder without OrthoRefine. *E. coli*, *S. enterica*, and *K. pneumoniae* each contributed one gene to HOG 346, while *P. aeruginosa* contributed four genes. OrthoRefine, with window size eight and synteny ratio 0.5, did not resolve HOG346; none of the four *P. aeruginosa* homologs could be classified as syntenous due to a lack of matches within the window. However, this HOG was resolved with the parameters we recommend for more distantly related genomes—window size 30 and synteny ratio 0.2, which identified the *P. aeruginosa* gene AFI95_RS32400 (transcription regulator *luxR* family) as the ortholog of *sdiA* in *E. coli*, *S. enterica*, and, by proxy, *K. pneumoniae* (Table 4). We speculate that the lack of synteny between the *K. pneumoniae* gene and any of the four genes of *P. aeruginosa* could stem from the fact that the syntenous genes between *E. coli*, *S. enterica*, and *P. aeruginosa* were motility genes, whereas *K. pneumoniae* is non-motile [50], and therefore not expected to contain these genes. Nevertheless, this conclusion is apparent only from the synteny analysis, whereas neither sequence similarity (Additional file 2: Table S3) nor the phylogenetic tree (Additional File 3, Figure S4) could differentiate an ortholog from the paralogs in the *P. aeruginosa* genome.

**Table 4:** Synteny ratio between genes for HOG 346

Genius species	Locus tag	Synteny ratio					
		*E. coli*	*S. enterica*	*Pseudomonas aeruginosa*			
		b1916	STM1950	AFI95_RS32400	AFI95_RS29375	AFI95_RS07465	AFI95_RS28195
*E. coli*	b1916	–	–	0.22	0.00	0.00	0.00
*S. enterica*	STM1950	0.9	–	0.33	0.03	0.00	0.00
*K. pneumoniae*	N559_RS09495	0.86	0.83	0.00	0.00	0.00	0.00

The four Gammaproteobacteria genomes were analyzed with OrthoRefine (window size = 30; synteny ratio = 0.2)

### Actinomycetota dataset

**Fig. 7:**
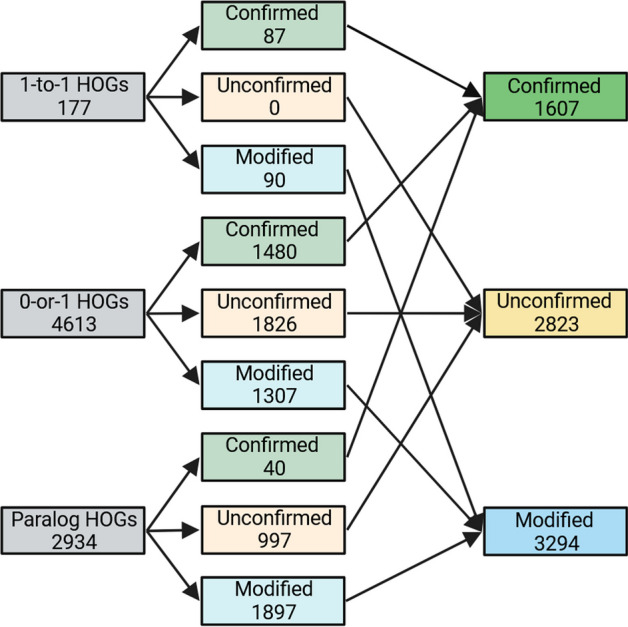
Summary statistics for the sixteen Actinomycetota genomes. The genomes were analyzed with OrthoRefine (window size = 8; synteny ratio = 0.5). See legend to Fig. 3

This dataset of sixteen arbitrarily selected genomes from the phylum Actinomycetota (Table 5) further increased the evolutionary distances among the analyzed genomes. Of the HOGs identified by OrthoFinder, only 2% were 1-to-1, 60% were 0-or-1, and 38% were paralogous. OrthoRefine modified 65% of the HOGs with paralogs; additionally, OrthoRefine confirmed 49% and 32% of the 1-to-1 and 0-or-1 HOGs (Fig. 7).

**Table 5:** Species names and Refseq accession for the sixteen Actinomycetota genomes

Genus species	Refseq accession
*Cryptobacterium curtum*	GCF_000023845.1
*Olsenella timonensis*	GCF_900119915.1
*Olsenella uli*	GCF_000143845.1
*Eggerthella lenta*	GCF_021378605.1
*Egibacter rhizosphaerae*	GCF_004322855.1
*Egicoccus halophilus*	GCF_004300825.1
*Denitrobacterium detoxificans*	GCF_001643775.1
*Rubrobacter xylanophilus*	GCF_000014185.1
*Rubrobacter tropicus*	GCF_011492945.1
*Rubrobacter marinus*	GCF_011492965.1
*Acidimicrobium ferrooxidans*	GCF_000023265.1
*Streptomyces fradiae*	GCF_008704425.1
*Streptomyces griseus*	GCF_000010605.1
*Streptomyces avermitilis*	GCF_000009765.2
*Acidothermus cellulolyticus*	GCF_000015025.1
*Actinomyces oris*	GCF_016127955.1

**Case study 4: HOG 402 – (PknB)** HOG 402 is comprised of 23 genes from the sixteen species, which are all annotated as encoding proteins of the kinase B (PknB) family – which contains penicillin-binding proteins (PBP) and serine/threonine kinases (STKP) characterized by presence of a serine/threonine kinase-associated domain (PASTA). The *pknB* gene is essential in *Mycobacterium tuberculosis* [51, 52], where it controls cell division and cell wall synthesis [53]; however, *pknB* was found to be not essential in *Streptomyces coelicolor,* where it is thought to be involved in the development cycle and antibiotic production [54]. In PBPs, the function of the PASTA domain appears to be species specific [55], and there is a lack of consensus on its exact function [56]. In STKPs, the PASTA domain is thought to bind peptidoglycan and β lactam (penicillin group antibiotics) [57].

**Fig. 8:**
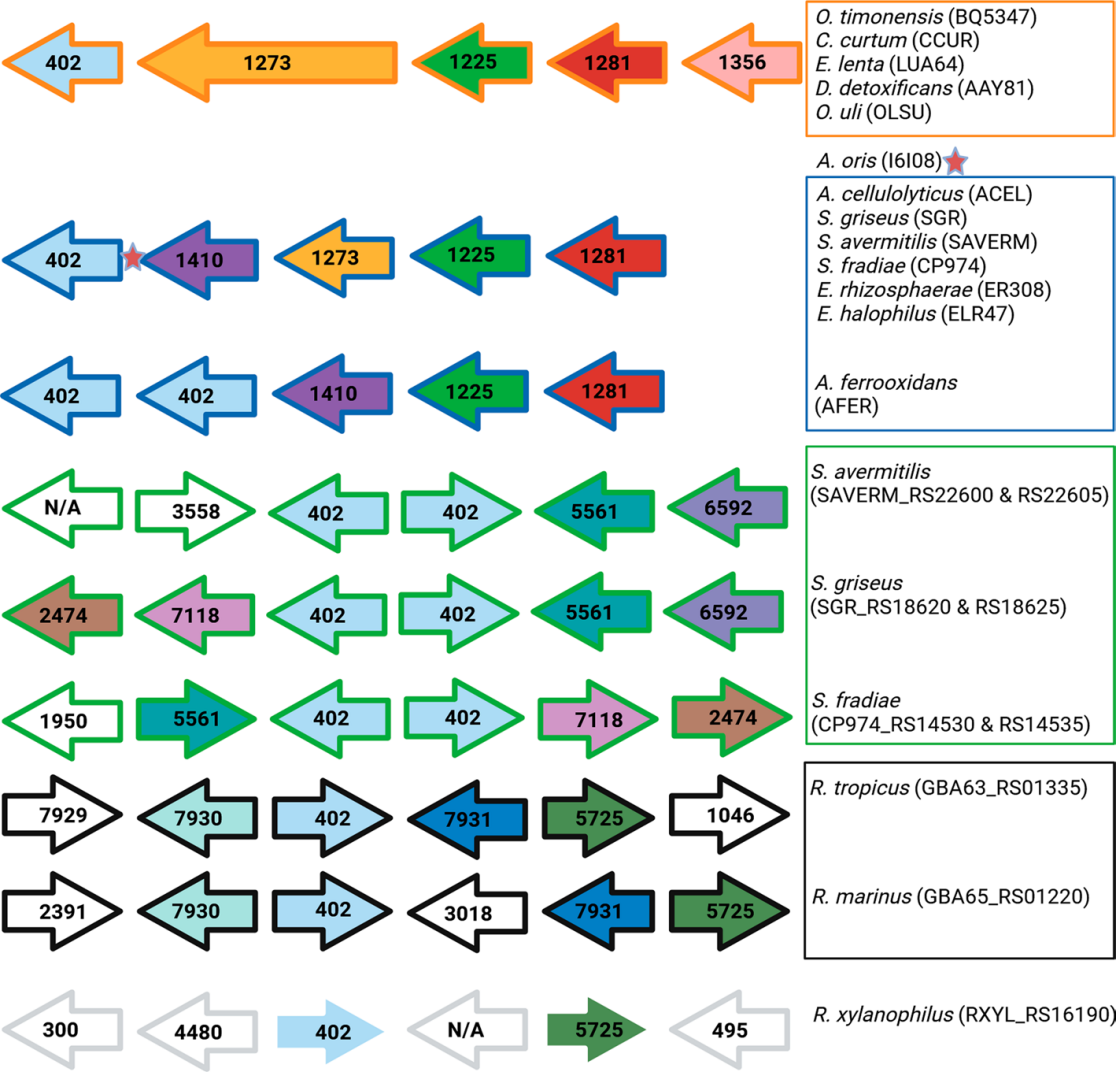
Synteny analysis of HOG 402. Matched colored arrows represent the same HOG number, which have been placed inside the arrows; white arrows denote genes with no match from the same HOG within the window, arrows containing N/A were not assigned to a HOG. The Actinomycetota operons of *pknB* have been divided based on SOG assignment and their edges color coded (SOG 402.0 orange, 402.1 blue, 402.2 green, or 402.3 black). Additional matches within the window have been omitted from the figure as the focus was on the operon. Due to an additional STPK (red star), assigned to HOG 400, between the STPK and the PBP, *A. oris* was not assigned to any SOG but otherwise has the same operon as SOG 402.1

OrthoRefine split the HOG into four SOGs (Fig. 8). SOG 402.0 contained the genes from *O. timonensis*, *O. uli*, *D. detoxificans*, *C. curtum*, and *E. lenta*. SOG 402.1 included two genes from *A. ferrooxidans* and one member each from *E. rhizosphaerae*, *E. halophilus*, *A. cellulolyticus*, *S. fradiae*, *S. avermitilis*, and *S. griseus;* the two genes from *A. ferrooxidans* are in tandem next to each other, which prevents them from being differentiated by synteny. SOG 402.2 contained similar pairs of tandem paralogs from the *Streptomyces* genera. SOG 402.3 included genes from *R. tropicus* and *R. marinus*, whereas the genes from *R. xylanophilus* and *A. oris* lacked the required synteny to be assigned to any SOG.

The members of SOG 402.0 and SOG 402.1 were identified as members of a previously identified operon from *Mycobacterium* [52, 53, 58] and *Streptomyces* [55]. The members of SOG 402.2 are not organized in the same operon, which provided further evidence for placing these genes into their own SOG (see Additional file 3: Figure S5 for additional details on supporting these SOG groupings).

### *Saccharomyces* dataset

**Fig. 9:**
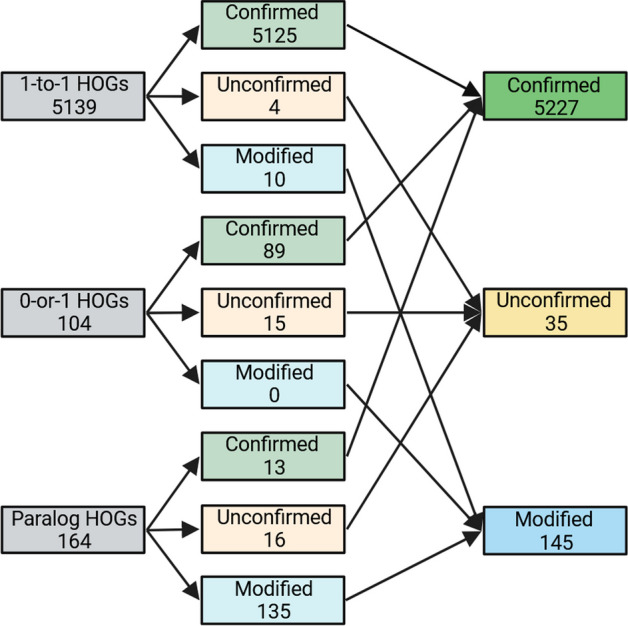
Summary statistics for the sixteen *Saccharomyces* genomes. The genomes were analyzed with OrthoRefine (window size = 8; synteny ratio = 0.5). See legend to Fig. 3

We evaluated three Saccharomyces genomes for orthologs to test OrthoRefine’s performance on eukaryotic genomes: *S. mikatae* (GCF_947241705.1), *S. cerevisiae* (GCF_000146045.2), and *S. kudriavzevii* (GCF_947243775.1). As expected, due to the small evolutionary distance between the three *Saccharomyces* genomes, 95% of OrthoFinder’s HOGs were 1-to-1, 2% were 0-or-1, and 3% were paralogous. OrthoRefine modified 82% of the paralogous HOGs and confirmed 99% of 1-to-1 HOGS and 85% of the 0-or-1 HOGs (Fig. 9).

**Fig. 10:**
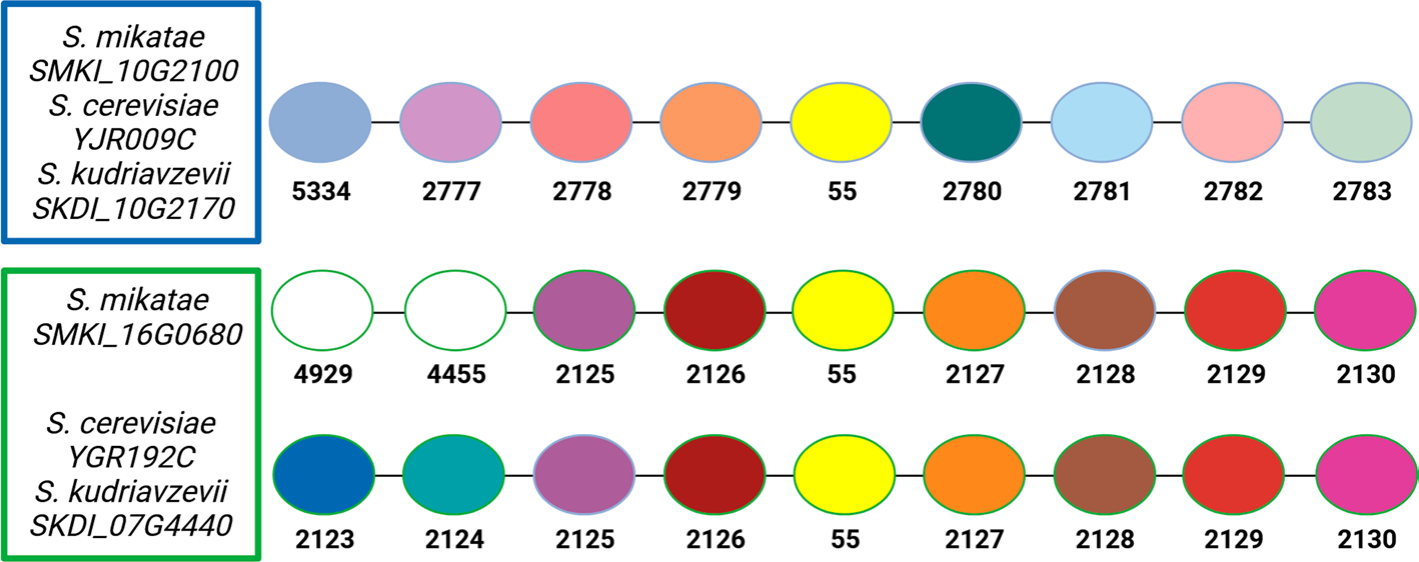
Synteny analysis of HOG 55. Matched colored circles represent genes assigned to the same HOG (shown below each circle); white circles denote genes with orthologs in other genomes located outside the displayed window. Blue and green boxes mark the two SOGs derived from HOG55

**Case Study 5: HOG 55—(glyceraldehyde-3-phosphate dehydrogenase).** HOG 55 is composed of two genes from each of the three genomes—annotated as encoding glyceraldehyde-3-phosphate dehydrogenase, either *tdh2* or *tdh3*—which are known paralogs [59]. OrthoRefine split the HOG into two SOGs, correctly separating the members of the two groups: SOG55.0, the *tdh2* group, was comprised of SMKI_10G2100, YJR009C, & SKDI_10G2170, whereas SOG55.1, the *tdh3* group, was comprised of SMKI_16G0680, YGR192C, & SKDI_07G4440 (Fig. 10). The BLAST e-value and percent identity mostly agreed with these groupings (Additional file 2: Table S4); however, SKDI_07G4440 was the best match for both *S*. *cerevisiae* genes, which led to a failure in correct ortholog assignment for the two paralogs in *S. cerevisiae* based on sequence similarity alone. It has previously been reported that orthologs sometimes have a lower percent identity compared to their paralogs [58, 60]. This result shows that synteny can, at least in some instances, resolve such discrepancies in sequence divergence. The phylogenetic analysis mostly supported the synteny groupings; however, similar to the BLAST e-values, there was a lack of support to tell where to group the genes from *S. kudriavzevii* (Additional file 3: Figure S6).

## Conclusion

We developed OrthoRefine, a standalone program that automates refinements of ortholog identification by evaluating gene synteny. OrthoRefine is designed to mimic the desirable properties of OrthoFinder, namely ease-of-use (no dependencies on additional software, a simple input, and support scripts to download data or create summary statistics), automation, and speed. We expect OrthoRefine to be most beneficial when the desired orthologous relationship is 1-to-1 (i.e., no paralogs). The value of synteny for ortholog identification has been demonstrated in previous studies [31, 60–63], but in the absence of easy-to-use tools to identify syntenous orthologs automatically, such studies have been time-intensive and generally limited in their scope. This work further demonstrates how the use of synteny, automated in OrthoRefine, can enhance ortholog identification by analyzing different data sets and groups separated by different distances. In addition to confirmation of OrthoRefine’s ability to increase specificity and functional ortholog identification via the community benchmarking tool, detailed investigation of several cases by manual inspection of sequence alignments, phylogenetic trees, and operon structures provided additional independent support for OrthoRefine’s results.

### Supplementary Information

**Additional file 1.** Bash commands used to generate benchmark dataset.

**Additional file 2.** Tables of BLAST e-values and percent identity.

**Additional file 3.** Phylogenetic trees and operon diagrams with supporting text.

**Additional file 4.** Bash and R commands used to generate phylogenetic trees.

## Data Availability

Datasets analyzed for this study are available at OrthoRefine’s GitHub repository: https://github.com/jl02142/OrthoRefine/tree/main/pub_data.

## Abbreviations

ABC: Adenosine triphosphate (ATP)-binding cassette
AMNOG: Average max number of orthologous genes
BLAST: Basic Local Alignment Search Tool
EC: Enzyme classification
GO: Gene ontology
HOG: Hierarchical orthogroups
ISNCY: Insertion sequence family not classified yet
MCL: Markov clustering
NCBI: National Center for Biotechnology Information
PASTA: Penicillin-binding protein and serine/threonine kinase associated domain
Pkn: Protein kinase
PBP: Penicillin-binding protein
RBH: Reciprocal best hit
RF: Robinson-Foulds
SOG: Syntenic ortholog group
STKP: Serine/threonine kinase
