# Supplementary material for: OrthoRefine: automated enhancement of prior ortholog identification via synteny
Source: BMC Bioinformatics. 2024 Apr 25;25:163. doi: 10.1186/s12859-024-05786-7 (PMC11044567; doi:10.1186/s12859-024-05786-7)
Supplement: Supplementary file 1 — Additional file 1. Bash commands used to generate benchmark dataset. [file 12859_2024_5786_MOESM1_ESM.docx]

**Bash commands and input files used to generate benchmark dataset**

**----------------------------------------------------------------------------**

# Download data files from Benchmark website

<ftp://ftp.ebi.ac.uk/pub/databases/reference_proteomes/previous_releases/qfo_release-2020_04_with_updated_UP000008143/QfO_release_2020_04_with_updated_UP000008143.tar.gz>

# move benchmark files to own directory, note you have to change file path where you want to move files

ls *.fasta | grep -v "DNA" | grep -v "addit" | xargs -I {} mv {} ../../benchmark_to_submit/bacteria/

# need to change fasta header line OrthoRefine’s pattern matching

for thing in *.fasta; do sed 's/^>..|/>/' $thing | sed 's/|/ |/' > temp.fasta; mv temp.fasta $thing; done

# Use README file to look up GCF accession for benchmarking dataset

# Included at the end of this file is a copy/paste file with input for using download script and input with GCF, GCA, UP, and species name

# Download GCF using OrthoRefine’s download script

./download_ft_fafiles “$file_name”

# Download GCA files manually

<https://ftp.ncbi.nlm.nih.gov/genomes/all/GCA/>

# Rename feature table file so they don’t interfere with OrthoRefine when running benchmark on their data

for thing in *feature_table.txt; do mv $thing "${thing%%?????????????????}ft.txt"; done

# Compile support script convert_locus_tag_to_prot_id.cpp as convert_locus_tag_to_prot_id.exe

g++ convert_locus_tag_to_prot_id.cpp -o convert_locus_tag_to_prot.exe

# Perform conversion on REFSEQ feature table to change to uniport ID

for thing in GCF*_ft.txt; do thing2="GCA${thing##???}"; thing3="${thing2%_*}"; thing4="${thing3%_*}".txt; ./convert_locus_tag_to_prot.exe $thing $thing4; done

# rename fasta file to match OrthFinder output to OrthoRefine input, note detailed input file which is included at the end of this file

cat detailed_input_all_bacteria_benchmark.txt | awk '{print $3"* "$1".fasta"}' > move_list

while read line; do mv $line; done < move_list

# Move .faa files to different directory so OrthoFinder doesn’t use them

mv *.faa /path/to/dir

# Run OrthoFinder

/path/to/OrthoFinder/./orthofinder -f ./

# use dos2unix on OrthoFinder output

dos2unix N0.tsv

# Generate OrthoRefine results, also get lines that are extra to remove from OrthoFinder output to match OrthoRefine’s dataset which has missing data (difference between NCBI and uniprot as NCBI is 2023 data and uniprot is 2020, can’t find NCBI 2020 data backup)

./OrthoRefine.exe -input input_all_bacteria_benchmark.txt -OF_file N0.tsv -window_size 8 -synteny_ratio 0.5 -benchmark 1 -run_all 1 -print_all_orthofinder 1 | grep "prod" | cut -d " " -f3 > list_to_remove_from_orthofinder_as_no_ft_match.txt

# Convert OrthoFinder output file to 2 columns for submission

./convert_orthofinder_out_to_benchmark_submit.exe N0.tsv > 16_bac_orthofinder.tsv

# remove extra lines of data from OrthoFinder’s output that are not in the feature table to submit to benchmark. Keeps dataset the same between OrthoFinder and OrthoRefine. Slow but works

while read line; do sed -i "/$line/d" ./16_bac_orthofinder.tsv; done < list_to_remove_from_orthofinder_as_no_ft_match.txt

# Uniq to remove duplicates from OrthoRefine output

cat OrthoRefine_outfile | sort | uniq > sorted_OrthoRefine_outfile

# Submit to Ortholog benchmarking service

------------------------------------------------------------

# input_all_bacteria_benchmark.txt

GCF_000195955.2

GCF_000006765.1

GCF_000008545.1

GCF_000008725.1

GCF_000203835.1

GCF_000092565.1

GCF_000005845.2

GCF_000008805.1

GCF_000008565.1

GCF_000011365.1

GCF_000009725.1

GCF_000018865.1

GCF_000009045.1

GCF_000011385.1

GCF_000008625.1

GCF_000008525.1

GCF_000007325.1

GCF_000196115.1

GCF_000007985.2

GCF_000027325.1

GCF_000021645.1

GCF_000011065.1

GCF_000020985.1

# detailed_input_all_bacteria_benchmark.txt

GCF_000195955.2 GCA_000195955.2 UP000001584 Mycobacterium tuberculosis strain H37RV

GCF_000006765.1 GCA_000006765.1 UP000002438 Pseudomonas aeruginosa PAO1

GCF_000008545.1 GCA_000008545.1 UP000008183 Thermotoga maritima MSB8

GCF_000008725.1 GCA_000008725.1 UP000000431 Chlamydia trachomatis (strain D/UW-3/Cx)

GCF_000203835.1 GCA_000203835.1 UP000001973 Streptomyces coelicolor (strain ATCC BAA-471 / A3(2) / M145) surpressed by REFSEQ

GCF_000092565.1 GCA_000092565.1 UP000001408 Leptospira interrogans serogroup Icterohaemorrhagiae serovar Lai (strain 56601)

GCF_000005845.2 GCA_000005845.2 UP000000625 Escherichia coli (strain K12)

GCF_000008805.1 GCA_000008805.1 UP000000425 Neisseria meningitidis serogroup B (strain MC58)

GCF_000008565.1 GCA_000008565.1 UP000002524 Deinococcus radiodurans (strain ATCC 13939 / DSM 20539 / JCM 16871 / LMG 4051 / NBRC 15346 / NCIMB 9279 / R1 / VKM B-1422)

GCF_000011365.1 GCA_000011365.1 UP000002526 Bradyrhizobium diazoefficiens (strain JCM 10833 / IAM 13628 / NBRC 14792 / USDA 110)

GCF_000009725.1 GCA_000009725.1 UP000001425 Synechocystis sp. (strain PCC 6803 / Kazusa)

GCF_000018865.1 GCA_000018865.1 UP000002008 Chloroflexus aurantiacus (strain ATCC 29366 / DSM 635 / J-10-fl)

GCF_000009045.1 GCA_000009045.1 UP000001570 Bacillus subtilis (strain 168)

GCF_000011385.1 GCA_000011385.1 UP000000557 Gloeobacter violaceus (strain ATCC 29082 / PCC 7421)

GCF_000008625.1 GCA_000008625.1 UP000000798 Aquifex aeolicus (strain VF5)

GCF_000008525.1 GCA_000008525.1 UP000000429 Helicobacter pylori (strain ATCC 700392 / 26695) (Campylobacter pylori) surpressed by REFSEQ

GCF_000007325.1 GCA_000007325.1 UP000002521 Fusobacterium nucleatum subsp. nucleatum (strain ATCC 25586 / CIP 101130 / JCM 8532 / LMG 13131) surpressed by REFSEQ

GCF_000196115.1 GCA_000196115.1 UP000001025 Rhodopirellula baltica (strain DSM 10527 / NCIMB 13988 / SH1)

GCF_000007985.2 GCA_000007985.2 UP000000577 Geobacter sulfurreducens (strain ATCC 51573 / DSM 12127 / PCA)

GCF_000027325.1 GCA_000027325.1 UP000000807 Mycoplasma genitalium (strain ATCC 33530 / G-37 / NCTC 10195)

GCF_000021645.1 GCA_000021645.1 UP000007719 Dictyoglomus turgidum (strain Z-1310 / DSM 6724)

GCF_000011065.1 GCA_000011065.1 UP000001414 Bacteroides thetaiotaomicron (strain ATCC 29148 / DSM 2079 / NCTC 10582 / E50 / VPI-5482)

GCF_000020985.1 GCA_000020985.1 UP000000718 Thermodesulfovibrio yellowstonii (strain ATCC 51303 / DSM 11347 / YP87)
