# Supplementary material for: OrthoRefine: automated enhancement of prior ortholog identification via synteny
Source: BMC Bioinformatics. 2024 Apr 25;25:163. doi: 10.1186/s12859-024-05786-7 (PMC11044567; doi:10.1186/s12859-024-05786-7)
Supplement: Supplementary file 2 — Additional file 2. Tables of BLAST e-values and percent identity. [file 12859_2024_5786_MOESM2_ESM.docx]

**Supplementary tables**

Table S1. Orthofinder's e-values and percent identity for two genes from E. coli, b0652 & b3271, as BLASTed against the other members of HOG19 from E. albertii, E. fergusonii, and E. marmotae. Bolded values are the lowest e-value and highest percent identity from b0652 or b327. b4106 & b4096 were ommitted due to high e-value (1.6e-35 & 2.6e-18) and low percent identity (39.1 & 31.2). HVX45_RS02390, HVX45_RS04025, & HVX45_RS09410 were omitted for the same reason (best e-value with E. coli. = 5.9e-56, best percent identity = 51.08).

| HOG 19 | | | | |
| --- | --- | --- | --- | --- |
| *E. coli*  b0652 | |  | *E. coli*  b3271 | |
| e-value | % identity | Gene to be BLAST against | e-value | % identity |
| **1.0e-131** | **98.8** | JRC41_RS15115  (*E. albertii*) | 2.1e-82 | 60.6 |
| **4.4e-133** | **100.0** | HVX45_RS07420  (*E. fergusonii*) | 2.8e-82 | 60.2 |
| **4.2e-133** | **100.0** | GV529_RS05870  (*E. marmotae*) | 2.7e-82 | 59.8 |
| 3.7e-79 | 60.2 | HVX45_RS11505  (*E. fergusonii*) | **1.1e-142** | **98.0** |
| 1.3e-78 | 60.2 | GV529_RS14465  (*E. marmotae*) | **2.3e-142** | **97.6** |

Table S2. Orthofinder's e-values and percent identity for two genes from E. coli, b2244 & b3411, as BLASTed against the other members of HOG21 from E. albertii, E. fergusonii, and E. marmotae. Bolded values are the lowest e-value and highest percent identity. HVX45_RS22925 had no reported best match within the HOG and had a very different length of 68 amino acids vs. the other member’s average length of 305 amino acids.

| HOG 21 | | | | |
| --- | --- | --- | --- | --- |
| *Escherichia coli*  b2244 (*rpnE*) | |  | *Escherichia coli*  b3411 (*rpnA*) | |
| e-value | % identity | Gene to be BLAST against | e-value | % identity |
| **2.2e-155** | **89.3** | JRC41_RS07400  (*E. albertii*) | 3.3e-100 | 57.9 |
| **3.9e-163** | **90.9** | HVX45_RS21485  (*E. fergusonii*) | 1.5e-98 | 65.2 |
| **5.9e-161** | **92.2** | GV529_RS12150  (*E. marmotae*) | 4.4e-100 | 58.1 |
| 4.7e-100 | 60.7 | HVX45_RS12120  (*E. fergusonii*) | **9.0e-157** | **93.5** |
| 5.1e-96 | 57.1 | GV529_RS10930  (*E. marmotae*) | **3.9e-149** | **86.3** |
| N/A | N/A | HVX45_RS22925  (*E. fergusonii*) | N/A | N/A |

Table S3. BLAST percent identity, reported by OrthoFinder, between genes for HOG 346 of the four Gammaproteobacteria genomes (E. coli, S. enterica, K. pneumoniae, & P. aeruginosa).

| BLAST percent identity | | | | | | |
| --- | --- | --- | --- | --- | --- | --- |
|  |  |  | P. aeruginosa | | | |
|  | b1916 | STM1950 | AFI95_RS32400 | AFI95_RS29375 | AFI95_RS07465 | AFI95_RS28195 |
| b1916 | - | - | 37.3 | 30.1 | 40.8 | 33.2 |
| STM1950 | 71.3 | - | 34.7 | 30.2 | 45.0 | 34.0 |
| N559_RS09495 | 65.8 | 66.7 | 25.7 | Not reported | 43.7 | 31.0 |

Table S4. Orthofinder's e-values and percent identity for two genes from S. cerevisiae, YJR009C & YGR192C, as BLASTed against the other members of HOG55 from S. mikatae & S. kudriavzevii. Bolded values are the lowest e-value and highest percent identity.

| HOG 55 | | | | |
| --- | --- | --- | --- | --- |
| *S*. *cerevisiae*  YJR009C | |  | *S*. *cerevisiae*  YGR192C | |
| e-value | % identity | Gene to be BLAST against | e-value | % identity |
| **1.5e-186** | **99.4** | SMKI_10G2100 | 6.4e-182 | 96.4 |
| 1.6e-183 | 97.3 | SKDI_10G2170 | 3.2e-181 | 95.8 |
| 8.7e-182 | 96.4 | SMKI_16G0680 | **2.4e-187** | **99.4** |
| **1.4e-184** | **97.9** | SKDI_07G4440 | **3.0e-182** | **96.4** |
