# Supplementary material for: OrthoRefine: automated enhancement of prior ortholog identification via synteny
Source: BMC Bioinformatics. 2024 Apr 25;25:163. doi: 10.1186/s12859-024-05786-7 (PMC11044567; doi:10.1186/s12859-024-05786-7)
Supplement: Supplementary file 3 — Additional file 3. Phylogenetic trees and operon diagrams with supporting text. [file 12859_2024_5786_MOESM3_ESM.docx]

**Supplementary figures**


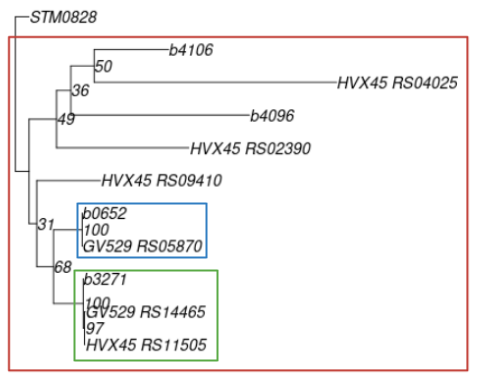
Figure S1. Phylogenetic tree generated by RAxML (see methods) of HOG 19 comprised of sequences from E. coli (prefix b), E. fergusonii (HVX45), E. marmotae (GV529), and E. albertii (JRC41). b0652 of E. coli is a representative of HVX45_RS07420 of E. fergusonii and JRC41_RS15115 of E. albertii. The best BLAST hit from S. enterica, STM0828, was used to root the tree. Boxes have been placed around Orthofinder’s grouping (red) and OrthoRefine’s groupings (blue or green). Node values are bootstrap support with n = 1000. There was bootstrap support (>70; [5]) for the blue and green groupings but not for additional groups.


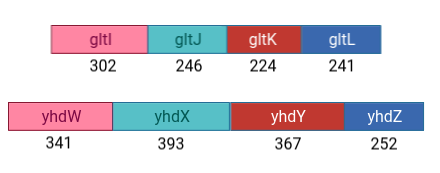
Figure S2. The gltIJKL and yhdWXYZ ABC transporter operons of the Escherichia genera. The product length is below each gene. The RNA gene, sroC, has been omitted from the gltIJKL operon for simplicity.

b0652, annotated as glutamate/aspartate ABC transporter ATP subunit gltl, is a member of the gltIJKL operon [1]. All members of SOG19.0 (b0652, JRC41_RS15115, HVX45_RS07420, & GV529_RS05870) were in the same operon in their respective genomes. Similarly, all members of SOG19.1 (b3271, HVX45_RS11505, & GV529_RS14465) were in the previously reported yhdWXYZ operon in their respective genomes [2, 3]. Members of both operons had the same product length within their operons (Figure S2), and the second and third sub-units (J/X & K/Y) followed the previously reported pattern of the first three of the more specific substrate-binding sub-units being about 30% smaller than their non-specific counterparts [3]. The exceptions, gltI & yhdW, were probably due to a frameshift in the yhd operon [2, 3].


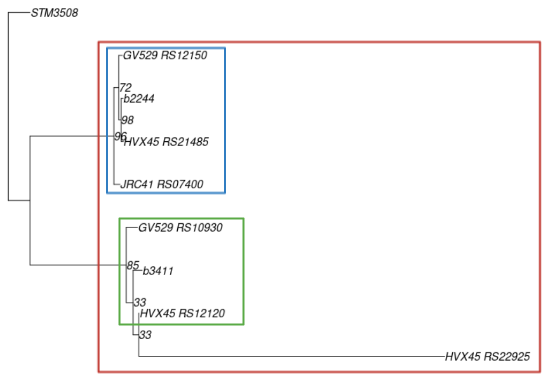
Figure S3. Phylogenetic tree of HOG 21 comprised of sequences from E. coli (prefix b), E. fergusonii (HVX45), E. marmotae (GV529), and E. albertii (JRC41). The best BLAST hit from S. enterica, STM3508, was used to root the tree. Boxes have been placed around Orthofinder’s grouping (red) and OrthoRefine’s groupings (blue or green). Node values are bootstrap support with n = 1000. There was bootstrap support (>70) for the blue and green groupings. HVX45_RS22925 lacked synteny support but had bootstrap support to be included in the green group; however, such a grouping would be non-monophyletic [4] and thus violated the species overlap method used to tell ortholog from paralog [6].


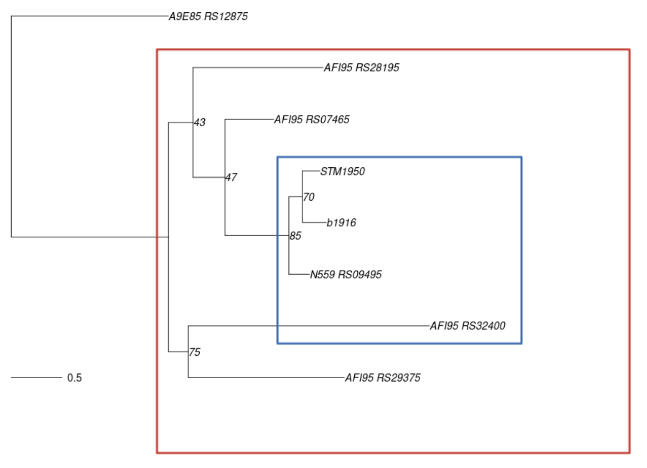
Figure S4. Phylogenetic tree of HOG 346 comprised of sequences from E. coli (b1916), K. pneumoniae (N559_RS09495), S. enterica (STM1950), and P. aeruginosa (prefix AFI95). The best BLAST hit from Legionella pneumophila, A9E85_RS12875, was used to root the tree. Boxes have been placed around OrthoFinder’s grouping (red) and OrthoRefine’s grouping (blue). There was a lack of bootstrap support (>70) for any of the four P. aeruginosa genes to be grouped with the genes from the other species. Node values are bootstrap support with n = 1000.


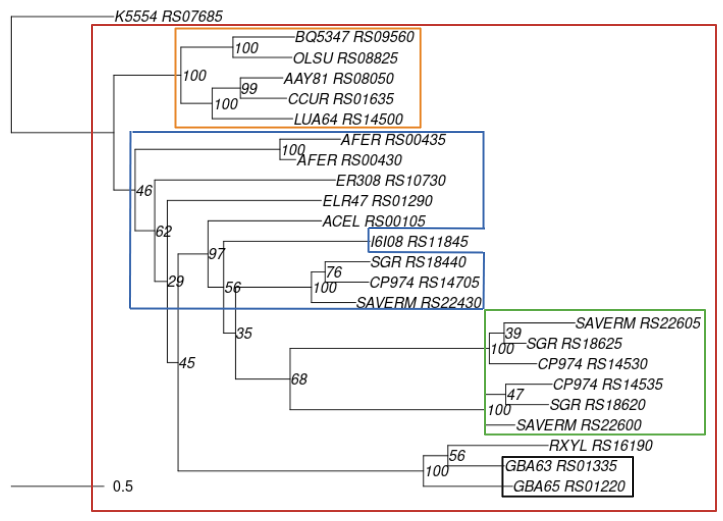


Figure S5. Phylogenetic tree of HOG 402 of the 16 Actinomycetota; the Gelria gene with the best BLAST hit was used to root the tree. OrthoFinder grouped all genes into a single HOG (red box), while OrthoRefine split the group into four SOGs (orange, blue, black, and green boxes). Node values are bootstrap support values with n = 1,000. There was a lack of bootstrap support (>70) to delineate the Streptomyces orthologs from paralogs using the species overlap method.

The operons for members of SOG 402.1 were consistently found to have a STPK adjacent to a PBP, FtsW, Stp1, and a forkhead-associated (FHA) domain; members of SOG 402.0 had a similar arrangement except the PBP and FtsW were fused (Figure 8). We could not detect an analogous operon containing the genes included in HOG402 from the *Rubrobacter* genomes. However, a manual inspection of the annotation for *R. xylanophilus, R. tropicus*, & *R. marinus* reveals the operon not with the *Rubrobacter* genes assigned to HOG402 but rather with those assigned to HOG401 (RXYL_RS00115, GBA63_RS00140, & GBA65_RS00120). We also detected a gene fusion or split - which has previously been shown to reduce the accuracy of ortholog identification [7] - between members of SOG 402.0 and SOG 402.1, which would explain why OrthoRefine split these groups into their own SOGs instead of combining them into a single SOG. Additionally, *A. oris* has an additional gene in its operon that was not present in SOG 402.0 or 402.1, which would explain why its gene failed to be grouped with any SOG.

In most *Streptomyces,* an STPK with four PASTA domains is positioned next to a PBP without the PASTA domain [8]. SAVERM_RS22430, SGR_RS18440, & CP974_RS14705 have four PASTA domains, while the tandem pairs of *Streptomyces* genes in SOG402.2 are annotated as having one PASTA domain. The operons of *Rubrobacter* revealed from the manual inspection contained an STPK gene with four PASTA domains adjacent to a PBP, like the *Streptomyces* group.


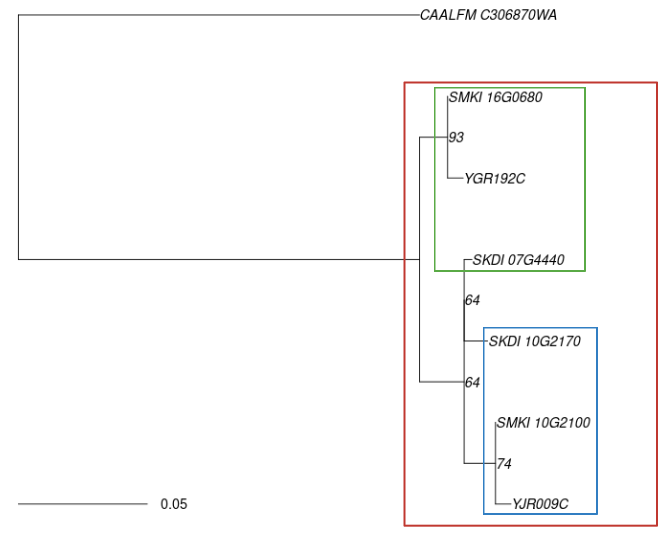
Figure S6. Phylogenetic tree of HOG 55 comprised of sequence from S. cerevisiae (YJR), S. mikatae (SMKI), and S. kudriavzevii (SKDI). The best BLAST hit from Candida albicans, CAALFM_C306870WA, was used to root the tree. Boxes have been placed around OrthoFinder’s grouping (red) and OrthoRefine’s groupings (blue or green). Node values are bootstrap support with n = 1000.

1. Miyakoshi M, Chao Y, Vogel J: **Cross talk between ABC transporter mRNAs via a target mRNA-derived sponge of the GcvB small RNA**. *EMBO J* 2015, **34**(11):1478-1492.

2. Walshaw DL, Poole PS: **The general L-amino acid permease of Rhizobium leguminosarum is an ABC uptake system that also influences efflux of solutes**. *Mol Microbiol* 1996, **21**(6):1239-1252.

3. Walshaw DL, Lowthorpe S, East A, Poole PS: **Distribution of a sub-class of bacterial ABC polar amino acid transporter and identification of an N-terminal region involved in solute specificity**. *FEBS Lett* 1997, **414**(2):397-401.

4. Hennig W: **Phylogenetic Systematics.** , 1st edn: University of Illinois Press; 1966.

5. Hillis DM, Bull JJ: **An Empirical Test of Bootstrapping as a Method for Assessing Confidence in Phylogenetic Analysis**. *Systematic Biology* 1993, **42**(2):182-192.

6. Huerta-Cepas J, Dopazo H, Dopazo J, Gabaldon T: **The human phylome**. *Genome Biol* 2007, **8**(6):R109.

7. Li G, Ma Q, Mao X, Yin Y, Zhu X, Xu Y: **Integration of sequence-similarity and functional association information can overcome intrinsic problems in orthology mapping across bacterial genomes**. *Nucleic Acids Res* 2011, **39**(22):e150.

8. Ogawara H: **Self-resistance in Streptomyces, with Special Reference to beta-Lactam Antibiotics**. *Molecules* 2016, **21**(5).
