# Supplementary material for: OrthoRefine: automated enhancement of prior ortholog identification via synteny
Source: BMC Bioinformatics. 2024 Apr 25;25:163. doi: 10.1186/s12859-024-05786-7 (PMC11044567; doi:10.1186/s12859-024-05786-7)
Supplement: Supplementary file 4 — Additional file 4. Bash and R commands used to generate phylogenetic trees. [file 12859_2024_5786_MOESM4_ESM.docx]

**Bash and R commands used to generate phylogenetic trees**

**-----------------------------------------------------------**

#Bash

# align with muscle 5

./muscle5.1.linux_intel64 -align “$sequence.fasta” -output “$aligned.fasta”

# build tree with raxml

raxml/standard-RAxML-master/./raxmlHPC-PTHREADS-AVX -T 10 -f a -m PROTGAMMAAUTO -p 12345 -x 12345 -o STM0828 -# 1000 -s “$aligned.fasta” -n “$hog_number_tree”

------------------------------------------------------------

#R

library(ape)

setwd() # set working directory

data1 <- read.tree(“RAxML_bipartitions.hog_number_tree”)

plot.phylo(data1, show.node.label = TRUE)

add.scale.bar()

------------------------------------------------------------

Tree figures have been rotated about nodes to place certain groups next to other groups.
